# Can subjective pain be inferred from objective physiological data? Evidence from patients with sickle cell disease

**DOI:** 10.1371/journal.pcbi.1008542

**Published:** 2021-03-11

**Authors:** Mark J. Panaggio, Daniel M. Abrams, Fan Yang, Tanvi Banerjee, Nirmish R. Shah

**Affiliations:** 1 Johns Hopkins University Applied Physics Laboratory, Laurel, Maryland, United States of America; 2 Department of Engineering Sciences and Applied Mathematics, Northwestern University, Evanston, Illinois, United States of America; 3 Department of Computer Science and Engineering, Wright State University, Dayton, Ohio, United States of America; 4 Department of Medicine, Duke University, Durham, North Carolina, United States of America; Brown University, UNITED STATES

## Abstract

Patients with sickle cell disease (SCD) experience lifelong struggles with both chronic and acute pain, often requiring medical interventMaion. Pain can be managed with medications, but dosages must balance the goal of pain mitigation against the risks of tolerance, addiction and other adverse effects. Setting appropriate dosages requires knowledge of a patient’s subjective pain, but collecting pain reports from patients can be difficult for clinicians and disruptive for patients, and is only possible when patients are awake and communicative. Here we investigate methods for estimating SCD patients’ pain levels indirectly using vital signs that are routinely collected and documented in medical records. Using machine learning, we develop both sequential and non-sequential probabilistic models that can be used to infer pain levels or changes in pain from sequences of these physiological measures. We demonstrate that these models outperform null models and that objective physiological data can be used to inform estimates for subjective pain.

## Author summary

Understanding subjective human pain remains a major challenge. If objective data could be used in place of reported pain levels, it could reduce patient burdens and enable the collection of much larger data sets that could deepen our understanding of causes of pain and allow for accurate forecasting and more effective pain management. Here we apply two machine learning approaches to data from patients with sickle cell disease, who often experience debilitating pain crises. Using vital sign data routinely collected in hospital settings including respiratory rate, heart rate, and blood pressure and amidst the real-world challenges of irregular timing, missing data, and inter-patient variation, we demonstrate that these models outperform baseline models in estimating subjective pain, distinguishing between typical and atypical pain levels, and detecting changes in pain. Once trained, these types of models could be used to improve pain estimates in real time in the absence of direct pain reports.

## 1 Introduction

Sickle cell disease (SCD) is a disorder that affects red blood cells and is associated with chronic pain as well as acute pain crises that often result in hospitalization [1]. During pain crises, patients experience severe pain that is managed with both opioid and non-opioid pain medication. Clinicians must therefore closely monitor their patients’ pain to determine appropriate dosing regimens and when patients are ready to be discharged. When monitoring pain, clinicians typically ask patients to rate their pain using a visual analogue scale (VAS) [2]. However, VAS pain reports are subjective and can exhibit significant inter-individual variation in pain scores reported in response to the same stimulus. These reports can also be influenced by a confluence of factors (e.g., mood, energy level, external emotional cues) that are unrelated to the patient’s physical condition.

Collecting subjective pain observations can be time-consuming and intrusive to patients. There are circumstances, such as when a patient is sleeping or heavily medicated, when it is impossible to collect this data. As such, it would be useful to be able to estimate pain levels from objective measurements that are easier to collect, cheaper, and more consistent. This would make it possible to track pain with high temporal resolution yielding richer datasets describing the evolution of pain over time. These datasets could lead to better understanding of the causes of pain and ultimately to models for forecasting pain.

Machine learning provides a powerful suite of tools for building predictive models from data and has led to significant progress on a wide variety of challenging scientific problems. In medicine, these methods are increasingly being used to develop personalized treatment strategies that have the potential to revolutionize patient care [3, 4]. Recent studies using linear discriminant analysis, support vector machines, neural networks and other machine learning methods for objective pain assessment with physiological signals such as electrical muscle activity, skin conductance level, and heart rate [5–7], facial expressions [8–10], activity and motion tracking [11, 12] and combinations of the above [13, 14] have yielded promising results. Here we consider the problem of estimating subjective pain scores in patients with sickle cell disease (SCD) using six objective physiological measures (vital signs) that are routinely collected.

We examined a dataset compiled from electronic medical records from 46 distinct SCD patients over a total of 105 hospitalizations at Duke Medical Center between January 1, 2014 and January 31, 2017. We used this data to train two types of probabilistic models, Gaussian naive Bayes (GNB) classifiers and hidden Markov models (HMMs), for inferring pain from the observed physiological measures. These models are capable of performing inference with partial observations and were used to ascertain the sequence of pain scores most likely to give rise to the observed physiological measures. We then compared these models to naive models that ignore the physiological measures in order to determine (a) whether the physiological measures contain useful information about the evolution of pain and (b) the extent to which that information is contained in the physiological measures themselves as compared to the sequential order of those observations.

## 2 Results

We investigated two types of models for estimating pain levels. HMMs use ordered physiological data and GNB models use unordered physiological data. The performance of these models was then compared to two null models (random and mode) that use no physiological data. These models are described in detail in Sec. 4.2.

We found that physiological data is indeed useful for estimating subjective pain. In three different pain classification tasks, described in Sec. 2.1, we found that the models that accounted for physiological data outperformed the baseline models that only considered the prevalence of each pain level. We also found that HMMs, which accounted for the sequential order of observations, outperformed the GNB models, which did not, in all three pain classification tasks.

**Fig 1:**
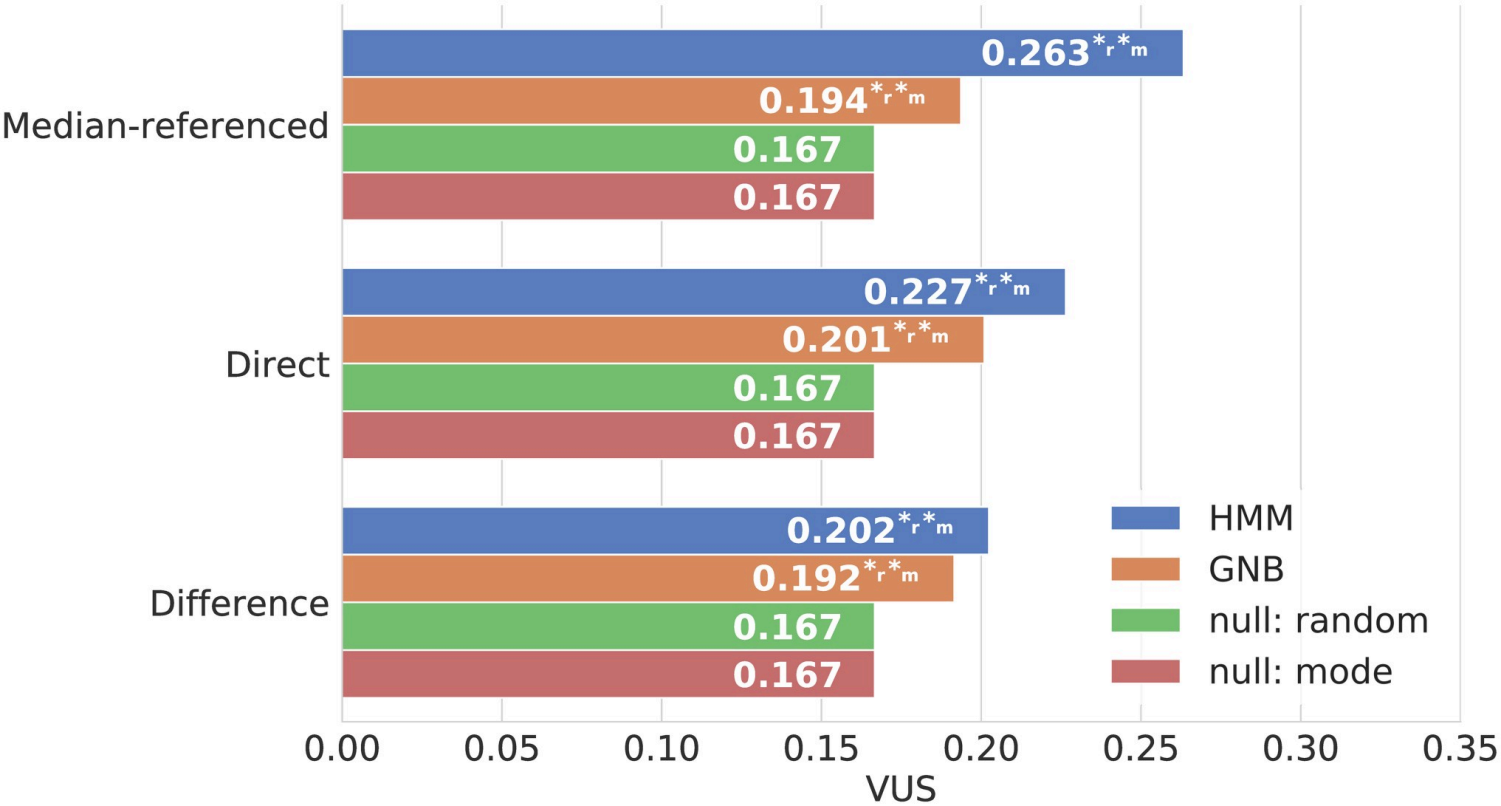
Model performance comparison. This figure displays the volume under receiver operating characteristic surface (VUS) for the HMM, GNB and null models (random, mode) on three classification tasks with three classes each. VUS can take on values between zero and one with zero indicating a classifier that is always wrong and one indicating a perfect classifier, so larger bars indicate better performance. Superscript *_r_ and *_m_ indicate the models that showed a statistically significant improvement in performance (at the *p* = 0.05 level) over the random and mode null models respectively.

The primary metric we use to assess performance of these models is the volume under the receiving operating characteristic surface (VUS), which can be interpreted as the probability that, given one randomly selected example from each class (e.g., one example with low pain, one with medium pain, and one with high pain), the model will correctly label each example [15, 16]. Fig 1 compares the performance of HMM and GNB models with the two null models which are only able to “guess” the correct labels 1/6 of the time. See Sec. 4.3 for further discussion of this evaluation metric.

We found that both the HMM and GNB models outperform this baseline, which indicates that physiological measures do contain information about the evolution of pain. This suggests that, with further refinement, these types of models could provide clinicians with information about patients’ pain levels in the absence of direct pain reports.

### 2.1 Classification problem formulation

We approached the problem of inferring pain from physiological measures as a classification problem, and explored three distinct variants. The definitions of the classes are summarized in Table 1.

**Table 1:** Class labels for three classification problem variants. *P*_*t*_ refers to the raw pain at time step *t*, *P*_*median*_ refers to the median pain for each patient during their inpatient stay, and Δ*P*_*t*_ = *P*_*t*+1_ − *P*_*t*_ refers to the change in pain since the last observation.

Problem	Direct	Median-referenced	Pain difference
Labels			
**0**	Low (0 ≤ *P*_*t*_ ≤ 3.333)	Median (*P*_*t*_ = *P*_*median*_)	No change (Δ*P*_*t*_ = 0)
**1**	Medium (3.333 < *P*_*t*_ ≤ 6.667)	Above Median (*P*_*t*_ > *P*_*median*_)	Increase (Δ*P*_*t*_ > 0)
**2 (or -1)**	High (6.667 ≤ *P*_*t*_ ≤ 10)	Below Median (*P*_*t*_ < *P*_*median*_)	Decrease (Δ*P*_*t*_ < 0)

In the first variant, we classified the raw pain scores *P*_*t*_ (where *t* refers to the time step) into three categories that correspond to low, medium, and high pain. We refer to this as “direct” pain classification.

In the second variant, we took a more personalized approach. Rather than assuming that the correspondence between physiological measures and pain was similar for all patients, we instead compared both the physiological measures and pain to the typical levels observed for each patient. This is motivated by the hypothesis that atypical levels of pain are characterized by atypical physiological measures (See S1 and S2 Figs). We therefore normalized the physiological measures by setting the means to zero and standard deviations to one for each patient, and assigned the pain scores to one of three categories: below the patient’s median pain level *P*_*median*_, at *P*_*median*_, and above *P*_*median*_. We refer to this second variant as “median-referenced” pain classification.

In the third variant, we predicted changes in pain, denoted Δ*P*_*t*_ = *P*_*t*+1_ − *P*_*t*_, rather than the pain itself. These changes between consecutive observations were assigned to one of three categories: decrease, no change, and increase. We refer to this third variant as “pain difference” classification.

**Fig 2:**
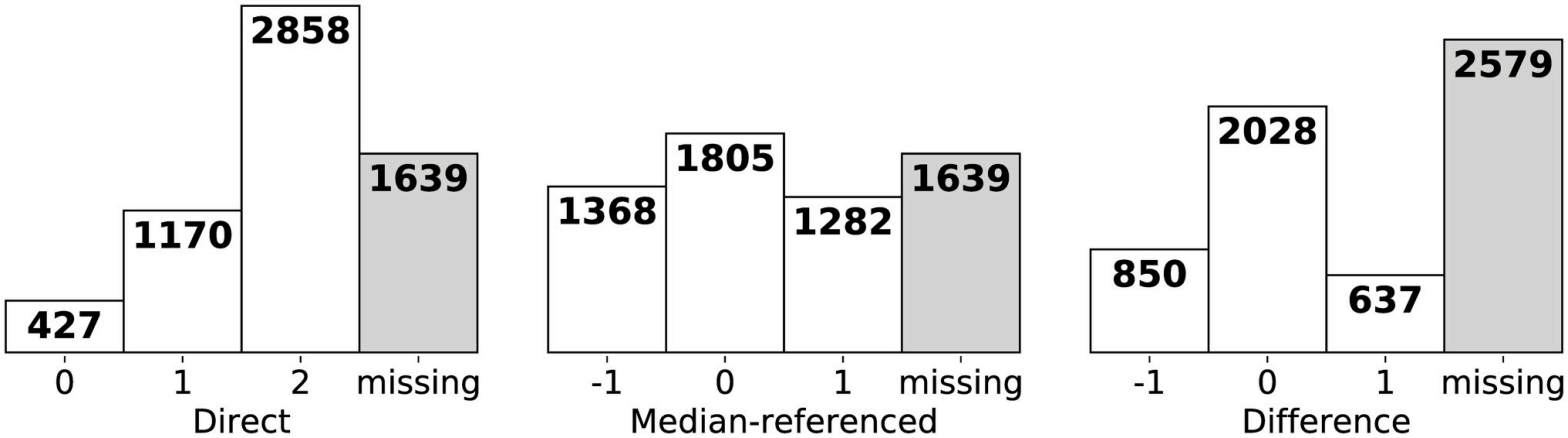
Class sizes for all three classification problem variants. There are more missing values for the difference classification problem due to the fact that Δ*P*_*t*_ = *P*_*t*+1_ − *P*_*t*_ is unknown when either *P*_*t*_ or *P*_*t*+1_ are unknown.

The numbers of observations in each class for all three variants are displayed in Fig 2.

### 2.2 Experiments

For each of the classification problems described in Section 2.1, we trained two probabilistic models, a Gaussian naive Bayes (GNB) classifier and a hidden Markov model (HMM). We trained these models in a semi-supervised fashion using 10-fold cross-validation, with data sequences randomly assigned to 10 subsets (folds). The models were optimized using nine folds and the performance was tested on the remaining fold. This was then repeated ten times for each of the possible divisions into training and testing sets. We chose 10 folds to balance the need for computational efficiency against the need for sufficiently large training and testing sets. We expect that similar results, albeit at substantially higher computational cost, could be obtained using leave-one-out cross-validation.

Rather than randomly distributing individual observations into folds, we assigned entire sequences of records for each hospital stay to folds to ensure that both the training and testing sets contained complete sequences. Because some patients had more than one hospital stay, data from the same patient did occasionally appear in both the training and testing sets, but the training and testing sets never contained data from the same hospital stay.

During testing, the models were used to infer the correct class labels for each observation given the physiological measures only. The performance was evaluated using standard classification metrics including: (1) VUS; (2) accuracy, the proportion of observations that were assigned the correct label; (3) precision, the number of true positives divided by the number of predicted positives averaged over all classes; (4) recall, the number true positives divided by the number of positive examples averaged over all classes; and (5) *F*_1_ score, the harmonic mean of precision and recall. The performance metrics reported here were computed on the testing fold. Further details about the data, models and training process, and evaluation metrics are provided in sections 4.1, 4.2 and 4.3 respectively.

We also generated two null models that ignore the physiological measures and predict based on the frequencies of the classes in the training data alone. In the “random” null model, labels were assigned randomly in proportion to the class frequencies in the training data. In the “mode” null model, all observations were assigned the label that was most common in the training data. These null models are of no clinical value. If the physiological measures provide meaningful information about pain, then one would expect the trained HMM and GNB classifiers to outperform the null models according to the metrics above. We therefore performed hypothesis tests to see if each of the trained models provided a statistically significant improvement over each null model (see Section 4.2 for details). The results of these experiments are displayed in Tables 2–4.

**Table 2:** Direct classification results for HMM, GNB and null models. The values in each cell represent the metric indicated by the column header. Precision, Recall and *F*_1_ score are computed using a macro average with uniform weights for each class. The values in parenthesis indicate the *p*-values associated with the random and mode null models respectively. When *p* < 0.05, the corresponding model performs better than the null model and that improvement is statistically significant at the 5% level.

Metric	VUS	Precision	Recall	*F*_1_ score	Accuracy
Model					
HMM	0.227 (< 0.001,< 0.001)	0.321 (0.955,< 0.001)	0.371 (< 0.001,< 0.001)	0.322 (0.689,< 0.001)	0.547 (< 0.001,1.000)
GNB	0.201 (< 0.001,< 0.001)	0.353 (< 0.001,< 0.001)	0.356 (< 0.001,< 0.001)	0.308 (0.990,< 0.001)	0.370 (1.000,1.000)
null: random	0.167	0.334	0.334	0.325	0.483
null: mode	0.167	0.211	0.333	0.256	0.632

**Table 3:** Median-referenced classification results for HMM, GNB and null models. The values in each cell represent the metric indicated by the column header. Precision, Recall and *F*_1_ score are computed using a macro average with uniform weights for each class. The values in parenthesis indicate the *p*-values associated with the random and mode null models respectively. When *p* < 0.05, the corresponding model performs better than the null model and that improvement is statistically significant at the 5% level.

Metric	VUS	Precision	Recall	*F*_1_ score	Accuracy
Model					
HMM	0.263 (< 0.001,< 0.001)	0.411 (< 0.001,< 0.001)	0.364 (< 0.001,< 0.001)	0.287 (1.000,< 0.001)	0.413 (< 0.001,0.018)
GNB	0.194 (< 0.001,< 0.001)	0.353 (< 0.001,< 0.001)	0.351 (< 0.001,< 0.001)	0.348 (< 0.001,< 0.001)	0.368 (< 0.001,1.000)
null: random	0.167	0.333	0.333	0.329	0.340
null: mode	0.167	0.134	0.333	0.190	0.402

**Table 4:** Difference classification results for HMM, GNB and null models. The values in each cell represent the metric indicated by the column header. Precision, Recall and *F*_1_ score are computed using a macro average with uniform weights for each class. The values in parenthesis indicate the *p*-values associated with the random and mode null models respectively. When *p* < 0.05, the corresponding model performs better than the null model and that improvement is statistically significant at the 5% level.

Metric	VUS	Precision	Recall	*F*_1_ score	Accuracy
Model					
HMM	0.202 (< 0.001,< 0.001)	0.360 (< 0.001,< 0.001)	0.354 (0.010,< 0.001)	0.347 (0.015,< 0.001)	0.501 (< 0.001,1.000)
GNB	0.192 (< 0.001,< 0.001)	0.359 (< 0.001,< 0.001)	0.361 (< 0.001,< 0.001)	0.344 (0.042,< 0.001)	0.401 (0.994,1.000)
null: random	0.167	0.333	0.333	0.330	0.421
null: mode	0.167	0.191	0.333	0.242	0.572

In the direct classification problem, both the HMM and GNB classifiers outperformed both null models in VUS and recall and they outperformed at least one of the null models in precision and *F*_1_ score. The mode prediction model had the highest accuracy due to the fact that the majority of pain reports corresponded to the high pain class in both the training and testing data. As a result, a trivial classifier that always predicts high pain can obtain a high degree of accuracy despite being of no clinical value. Both the HMM and GNB classifiers were trained by maximizing the log likelihood of the observed sequences of pain classes in the training data. As such, they assigned non-zero probability to minority classes. Improving the accuracy on the minority classes decreased the accuracy on the majority class resulting in lower accuracy overall, but better performance according to other metrics. These performance metrics are displayed in Table 2.

In median-referenced classification, we found that both the HMM and GNB models had statistically significant higher performance than both null models according to VUS, precision and recall. This lends support to the hypothesis that atypical pain levels correspond to atypical physiological data. However, the *F*_1_ score for the random guessing model was higher than the HMM. This is counter-intuitive due to the fact that *F*_1_ score is the harmonic mean of precision and recall and both the precision and recall exceed those of the null models. This observation can be explained by the fact that this metric is computed by first computing the harmonic mean of precision and recall on each class and fold before averaging the results with equal weights for each class. Zero precision or recall on one of the smaller classes will cause the corresponding *F*_1_ score to be zero which can have a dramatic effect on the averaged result. If instead the precision and recall were averaged over each class and fold first before computing the harmonic mean, then the the result would be higher for the HMM and GNB. The HMM also outperformed the null models in accuracy.

In difference classification we found that both the HMM and GNB model outperformed both null models in VUS, precision, recall and F_1_ score. Again the trivial mode model has higher accuracy due to the class imbalance. Notice that the F_1_ score is again lower than both precision and recall despite being a type of average. This behavior underscores the limitations of the *F*_1_ score when applied to multi-class classification problems with a class imbalance.

In S1 and S2 Tables, we report the confusion matrices for the HMM and GNB models. We found that these classifiers had the highest accuracy on the majority class and noticeably lower accuracies for minority classes in each of the three classification problems.

In S3–S5 Tables we also report the model parameters for the HMM and GNB models. These can be used to identify temporal trends as well as relationships between pain and the physiological measures. We found that the difference between classes was typically smallest for the blood pressure (Systol and Diastol) and temperature (Temp) suggesting that these features are less informative than the pulse (Pulse), respiratory rate (Resp) and oxygen saturation (SpO2).

## 3 Discussion

Overall, we found that both classifiers provided statistically significant improvements over null models according to a majority of metrics. This suggests that physiological measures contain information that can be used to improve pain classification and detection models. We also found that the HMM, which considers the sequence of observations, had higher performance than the GNB classifier, which treats observations as independent, in four out of the five metrics considered on all three classification problems. This indicates that the order of observations can also be used to provide additional performance improvements.

We found that the best performing model, according to VUS, was the median-referenced model. This can be attributed to the fact that it is more personalized than the other models. By accounting for how the pain and physiological measures differ from the typical levels experienced by each patient, the model is better able to identify deviations from the norm. In this paper, the personalization methods considered are relatively simple: they involve standardization relative to the mean physiological measures and median pain level for each patient. Alternatively, one might imagine training a separate model for each patient. Given a sufficiently large amount of data for each patient, one would expect such a personalized model to outperform the models presented here as long as the physiological response to pain remains consistent over time.

Here we focus on three-class problems, but it would be straightforward to adapt our approach to classification problems with additional classes in order to obtain more precise pain estimates. For example, one could assign the difference to five categories instead of three: no change, slight increase, large increase, slight decrease and large decrease. However, this would involve training models with additional parameters and such models would be prone to over-fitting unless larger datasets with more patients and finer temporal resolution were available.

Our work demonstrates the potential of machine learning methods for inferring pain from objective measurements, with clear improvement over trivial models. We hypothesize that the performance of the models could be improved by including additional features. For example, heart rate variability, galvanic skin response, and activity measurements from accelerometers can also serve as useful features for pain estimation [17]. These features are not included in electronic medical records, but can be recorded using commercially available fitness trackers. Ensemble models that fuse data from these sources with the standard vital signs discussed above would likely outperform the models presented here.

**Fig 3:**
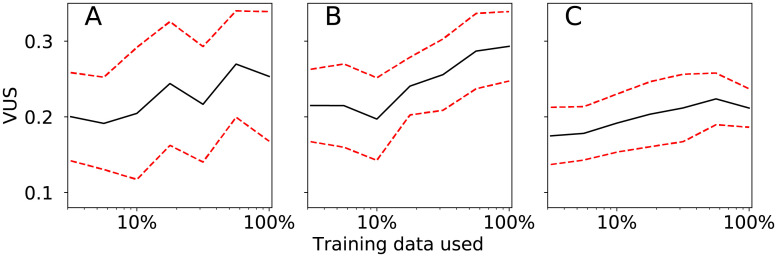
Learning curves. These plots display the volume under the receiver operating characteristic surface for the HMM classifier as the percentage of training data used to train the model varies. The full training set contained a randomly selected subset of up to 85% of the 4456 data points with known pain scores and the testing data contained the remaining 15%. The black (solid) curves correspond to the mean VUS averaged over 20 train-test splits. The red (dashed) curves correspond to the mean VUS ± one standard deviation to provide a measure of the variability in the performance. Panel A corresponds to the direct classification problem; panel B corresponds to the median-referenced classification problem; and panel C corresponds to the difference classification problem. The spacing on the horizontal axis is logarithmic.

The performance of these models could also be improved by training with larger datasets. In Fig 3, we display a learning curve for the HMM classifier in which the model was trained with only a fraction of the training data. As the fraction of the data increases, the VUS increases. The slopes of the trendlines *y* = *m* log(*x*) + *b* are positive and the slope is significantly greater than zero for the median-referenced model (with *p* = 0.008). The fact that the performance is still improving when the fraction reaches one suggests that performance of these classifiers would continue to improve with larger datasets. This data could be obtained either by increasing the size of the patient cohort or by increasing the observation frequency.

The HMM and GNB models discussed in this paper were selected because they are well-understood probabilistic models that are indistinguishable except for the sequential component. This allowed us to rigorously test whether physiological measures contain useful information about the evolution of pain and whether the sequence of observations contains useful trends for pain estimation. We hope that this proof of concept inspires further exploration with more powerful and flexible models that can be tuned for optimal performance.

The models presented here can be used either to make predictions about the pain class for each observation or to compute the probabilities for each class. These probabilities could be useful in a clinical setting by allowing doctors and nurses to determine whether a patient’s condition is likely to have changed. Prior strategies such as dosing and variations in the intervals of treatment have not been effective for patients with SCD. Prediction models could be valuable to inform treatment decisions such as when to adjust a patient’s medication. Importantly, although this study focused on patients with SCD admitted for pain, modeling to predict pain may be helpful for other patients admitted with pain. In addition, another clinically useful task would be pain forecasting: thus far the limited volume of pain data has made this a challenge, but using pain inferred from physiological data could make forecasting a realistic possibility.

## 4 Materials and methods

### 4.1 Data

Our results come from a dataset containing electronic medical records from both pediatric and adult patients with sickle cell disease that were admitted for vaso-occlusive crises at Duke Medical Center between January 1, 2014 and January 31, 2017. The dataset was limited to patients who were inpatient for at least 48 hours and required intravenous narcotics for treatment, such as via patient-controlled analgesia system. This included data from 46 unique patients over a total of 105 hospital visits. The demographics of this cohort of patients are typical of patients admitted with SCD pain in the US, however the conclusions we draw may not be applicable to outpatients or patients with SCD outside the US. We note that reference [18] also studied a subset of this data, using a different approach based on multiple imputation [19]. In this paper, in contrast, we use semi-supervised learning to develop models that can be used with incomplete data. As a result, our models could be used for real-time pain inference without a need for imputation of missing values.

We extracted records containing pain scores (PainScore) on an 11 point visual analogue scale along with six physiological measures: (i) peripheral capillary oxygen saturation (SpO2), (ii) systolic blood pressure (Systol), (iii) diastolic blood pressure (Diastol), (iv) heart rate (Pulse), (v) respiratory rate (Resp), and (vi) temperature (Temp). Each observation includes a unique patient identifier along with a timestamp indicating when the data was recorded. As such, the data for each patient can be viewed as a time series.

Time series models have been studied extensively [20]. Unfortunately, most time series models are designed for uniformly sampled observations that are completely observed and are therefore not directly applicable to these datasets which contain irregularly sampled pain scores and physiological measures and many missing features.

There are various ways to address the challenges of missing features and irregular sampling. We addressed the irregular sampling problem by aggregating the data into two-hour windows. When multiple observations were available during a given window, the observed features were averaged. This aggregation process decreases the total number of data points, but no data is lost and it results in uniformly spaced observations. Averaging has the added benefit of smoothing the fluctuations in the physiological measures and pain, and is justified as long as the changes in those features occur over sufficiently long time-scales. The percent of data present (non-missing) for each feature after this procedure is displayed in Table 5.

**Table 5:** Percentage of data present. Here values indicate the percentage of observations in the data that contain the indicated features.

Measure	Resp	Temp	Pulse	SpO2	Systol	Diastol	PainScore
Dataset							
Raw	54.6%	33.1%	43.6%	72.9%	33.4%	33.4%	52.7%
Aggregated	71.9%	51.5%	58.5%	82.3%	51.1%	51.1%	73.1%

Although aggregation into two-hour windows decreases the fraction of missing features in the data, it does not eliminate them entirely. We considered using imputation methods like fully conditioned specification (FCS) to fill in the remaining missing observations. However, these approaches rely on independent observations and therefore ignore sequential information during imputation. This means that trends in the physiological measures may not be captured during the imputation process. The imputation process is also computationally intensive and may not be suitable for real-time pain estimation. We therefore opted not to impute missing features and instead pursued probabilistic models that would allow us to perform inference conditioned on the available data.

After removing outliers that satisfied Diastol < 10 (severe hypotension), Systol > 180 (severe hypertension), and SpO2 < 80 (severe hypoxia) and were more than 5 standard deviations from the mean, we divided the data into 10 subsets, each with an approximately equal number of records (because there were 105 records total, subsets each contained either 10 or 11). Models were trained and tested using 10-fold cross-validation. In other words, the models were trained (i.e., parameters were estimated) using 9 subsets, and then tested (i.e., performance metrics were computed) on the remaining subset. This training and testing process was repeated 10 times, once for each possible subset arrangement, and the evaluation metrics on the testing folds were then averaged to provide an estimate of the generalization performance of the algorithm [21].

Probabilistic models typically require assumptions about the probability distribution of the features. For example, many models rely on the Gaussian naive Bayes assumption: that the features are independent conditioned on the class and are distributed according to a normal distribution. Even when these assumptions are not strictly satisfied, the resulting models often provide useful predictions [22].

In our case, we found that most of the physiological measures were weakly correlated (see Table 6 and S1 Fig) lending support for the independence assumption. Unfortunately, we found that they were not normally distributed. However, we circumvented this issue by using a non-parametric standardization method to transform the distributions to a normal distribution as follows:

1. Compute the empirical cumulative distribution function (ECDF) for each feature in the training set.
2. For each observed feature *x* in either the training or testing set, estimate the corresponding quantile *q* in the ECDF using linear interpolation.
3. Map the observed feature to the value *z*(*q*) that corresponds to quantile *q* in a standard normal distribution.

**Table 6:** Feature correlations. Here the values represent the correlation between the pair of features indicated by the row and column.

Measure	Resp	Temp	Pulse	SpO2	Systol	Diastol	PainScore
Resp		0.248	0.447	-0.054	0.113	0.192	-0.145
Temp			0.374	-0.085	0.048	0.093	-0.104
Pulse				-0.198	0.047	0.185	-0.083
SpO2					-0.110	-0.013	-0.033
Systol						0.585	0.017
Diastol							0.001

We applied this method to each of the ten cross-validation splits resulting in ten distinct standardized versions of the original data. This data was then used to train the models discussed below.

### 4.2 Models

We described the relationship between pain and the physiological measures using two probabilistic models: a Gaussian naive Bayes model and a hidden Markov model. Below we outline the assumptions of those models.

*Notation*: Let *y*_*t*_ denote the true pain class at time *t* and **x**_*t*_ denote the corresponding physiological measures. Following standard conventions, we use capital letters *X* and *Y* to denote random variables and lower case letters when referring to distributions.

For both classifiers, we were interested in estimating the probability distribution for the classes conditioned on the physiological measures,

$$\begin{array}{c} p(y_{t}|S_{T},\theta ), \end{array}$$

where ***θ*** denotes the set of parameters, *S*_*T*_ denotes the entire sequence of physiological measures **x**_1_, **x**_2_, …**x**_*T*_ and *T* is the length of the sequence for a given hospital stay. In the context of HMMs, the observed variables (the physiological measures) are referred to as emissions.

#### 4.2.1 Hidden Markov model

In an HMM, the state variable (the pain class) *y*_*t*_ is hidden and evolves according to a probabilistic process that possesses the Markov property,

$$\begin{array}{c} p\left(y_{t}|y_{t-1},y_{t-2},\ldots ,y_{1}\right)=p\left(y_{t}|y_{t-1}\right), \end{array}$$

i.e., the probability of transitioning to state *i* is independent of the history and depends only on the last state *j*. This process can therefore be fully specified by specifying a transition matrix *A* with *A*_*ij*_ = *p*(*Y*_*t*_ = *i*|*Y*_*t*−1_ = *j*).

We chose this type of model, which treats the evolution of pain as a random walk, due to the absence of consistent long-term trends in the observed pain (see S2 Fig) In the direct and median-referenced models, we found that the most common transitions were from state *i* to state *i*. In other words, the pain class remains constant most of the time. We also observed that transitions between adjacent pain classes occurred more frequently than non-adjacent classes. These frequencies were reflected in the transition matrices learned by the model (see S3 Table). In the difference classification problem, we observed that increases in pain were most often followed by decreases in pain and vice versa. Again, this was consistent with the learned transition matrices.

The datasets discussed here contain multiple sequences of states and emission variables, each of which corresponds to a single hospital stay. Below, we use the superscript ^(*n*)^ for *n* = 1, 2, …*N* to reference individual sequences when necessary, but suppress this superscript to simplify the notation when there is no ambiguity.

The emission variables are modeled as independent Gaussian random variables when conditioned on the hidden variable. In other words, the probability distribution of the physiological measures satisfies

$$\begin{array}{c} {\left(x_{t}\right)}_{k}|Y_{t}=i∼N\left(\mu_{ik},\sigma_{ik}^{2}\right), \end{array}$$

where *i* and *k* denote the particular class and physiological measure respectively and *μ*_*ik*_ and $\sigma_{ik}^{2}$ denote the corresponding mean and variance. The initial state in each sequence is modeled as a random variable that follows a multinomial distribution with prior probabilities *π*_0_, *π*_1_, and *π*_2_.

A fully specified three-state model with six independent emissions is therefore determined by a set of 48 parameters: three priors (*π*_*i*_), nine transition probabilities (*A*_*ij*_), 18 emission means (*μ*_*ik*_) and 18 emission variances ($\sigma_{ik}^{2}$). Training an HMM involves estimating these parameters from data. Typically this is achieved using maximum likelihood estimation (MLE), in which one selects the model parameters that are most likely to produce the observed sequences of emission variables. When the data is incomplete, MLE can be carried out using an expectation maximization method known as the Baum-Welch algorithm [23]. This algorithm is summarized as follows:

1. *Define initial parameter estimates **θ*** = (***π***, *A*, ***μ***, ***σ***^**2**^).
  We initialize these parameters by first assigning state labels to all observations with complete physiological measures using k-means clustering. Then, these estimated labels can be used to initialize the priors, by computing the proportion of sequences beginning in each state, and the transition matrix, by computing the proportions of transitions between each pair of states. The emission mean and variance for each class can be also computed using the standard statistical formulas.
2. *Compute the posterior probabilities for each state conditioned on the observed sequence of emissions using the forward-backward algorithm* [24].
  We compute the posterior probability *p*(*y*_*t*_|*S*_*T*_, ***θ***), and also compute the joint distribution for consecutive states *p*(*y*_*t*_, *y*_*t*+1_|*S*_*T*_, ***θ***). In agreement with standard notation, we define *γ*_*i*_(*t*) = *p*(*Y*_*t*_ = *i*|*S*_*T*_, ***θ***) and *ξ*_*ij*_(*t*) = *p*(*Y*_*t*_ = *i*, *Y*_*t*+1_ = *j*|*S*_*T*_, ***θ***).
3. *Update the parameter estimates using the posterior probabilities*.
  The priors can be estimated as follows:
  $$\begin{array}{c} \pi_{i}=\frac{1}{N}\sum_{n=1}^{N}\gamma_{i}^{\left(n\right)}\left(0\right) \end{array}$$
  where the sum is over all sequences. When the initial states are fully observed, *γ*_*i*_(0) ∈ {0, 1}, and this is equivalent to computing the proportion of sequences beginning in each state. When the initial states are partially observed, *γ*_*i*_(0) ∈ [0, 1], and this yields the expected proportion of sequences beginning in each state according to the posterior probabilities.
  The transition matrices can be estimated using:
  $$\begin{array}{c} A_{ij}=\frac{\sum_{n=1}^{N}\sum_{t=1}^{T-1}\xi_{ij}^{\left(n\right)}\left(t\right)}{\sum_{n=1}^{N}\sum_{t=1}^{T-1}\gamma_{i}^{\left(n\right)}\left(t\right)}. \end{array}$$
  For fully observed sequences, this involves computing the proportion of transitions from state *j* to state *i*. For partially observed sequences, this yields the expected proportion again using posterior probabilities.
  The means and standard deviations can be estimated using weighted formulas:
  $$\begin{array}{c} \mu_{ik}=\frac{\sum_{n=1}^{N}\sum_{t=1}^{T-1}\gamma_{i}^{\left(n\right)}\left(t\right){\left(x_{t}\right)}_{k}}{\sum_{n=1}^{N}\sum_{t=1}^{T-1}\gamma_{i}^{\left(n\right)}\left(t\right)}, \end{array}$$ (1)
  $$\begin{array}{c} \sigma_{ik}^{2}=\frac{\sum_{n=1}^{N}\sum_{t=1}^{T-1}\gamma_{i}^{\left(n\right)}\left(t\right)[{\left(x_{t}\right)}_{k}-\mu_{k}{]}^{2}}{\sum_{n=1}^{N}\sum_{t=1}^{T-1}\gamma_{i}^{\left(n\right)}\left(t\right)}. \end{array}$$ (2)
  As discussed above, the weights are posterior probabilities for each state. When the states are known, these reduce to the standard formulas for mean and variance.
4. *Repeat steps 2-3 are until the parameter estimates convergence*.
  We find that 100 iterations are generally sufficient.

In its original formulation, the Baum-Welch algorithm was designed for the case where the hidden states are never observed and the emission variables are completely observed. In the datasets studied here, some of the hidden states are known and many of the emission variables are missing. In this case, the forward backward procedure must be modified slightly, and the model is trained in a semi-supervised manner.

The first step of the forward-backward procedure involves computing the posterior probabilities for each state conditioned on the observed emissions using the current parameter estimates. This is given by

$$\begin{array}{c} p\left(Y_{t}=i|X_{t}=x,\theta \right)=\frac{p\left(X_{t}=x|Y_{t}=i,\theta \right)p\left(Y_{t}=i|\theta \right)}{\sum_{j=0}^{2}p\left(X_{t}=x|Y_{t}=j,\theta \right)p\left(Y_{t}=j|\theta \right)} \end{array}$$ (3)

which is derived from Bayes rule. The terms on the right hand side are straightforward to compute using the emission distributions, which are defined by the current parameter estimates ***θ***, and uniform priors *p*(*Y*_*t*_ = *i*|***θ***) = 1/3 for *i* = 0, 1, 2.

This step can be modified as follows. When no emissions are observed, we use uniform probabilities *p*(**X**_*t*_ = **x**|*Y*_*t*_ = *i*) = 1/3. When some but not all emissions are missing, *p*(**X**_*t*_ = **x**|*Y*_*t*_ = *i*, ***θ***) is computed by integrating over all possible values of the missing emissions. Since the emissions are independent, this distribution is just a product of Gaussians and one therefore only needs to multiply the densities that correspond to the observed emissions (when present). Finally, when the state variable is observed, we simply set the posterior probability *p*(*Y*_*t*_ = *i*|**X**_*t*_ = **x**, ***θ***) equal to one for the observed state and zero for all others.

Once the HMM has been trained, one can compute the sequence of hidden states that is most likely to produce the observed emissions using the Viterbi algorithm [25, 26].

#### 4.2.2 GNB model

The GNB model is a simplified version of the HMM. Rather then modeling three transitions between states, we treat the observations as if they are independent and identically distributed (IID). As with the HMM, we assume that the physiological measures are independent so that the *k*th emission is Gaussian when conditioned on the pain class.

The parameters of these distributions can be estimated in a semi-supervised fashion using a simplified version of the expectation maximization process used for HMMs. Rather than using the forward backward procedure to compute the posterior probabilities, we can instead exploit the IID assumption to compute them as follows:

$$\begin{array}{c} \gamma_{i}\left(t\right)=p\left(Y_{t}=i|S_{T},\theta \right)=p\left(Y_{t}=i|X_{t},\theta \right) \end{array}$$ (4)

where the term on the right hand side is given by Eq (3). Using this result, both the parameters and predictions of the model can be updated iteratively as follows:

1. *Initialize the parameters **θ*** = (***μ***, ***σ***^2^) *by computing the averages and variances of the labeled examples from each class*.
2. *Compute the posterior probabilities for fixed **θ** using* Eq (4).
3. *Update the parameters **θ** using these posterior probabilities and* Eqs (1) *and* (2).
4. *Repeat steps 2 and 3 until convergence*.

#### 4.2.3 Baseline models

Before training a sophisticated statistical or machine learning model like an HMM, it is useful to consider an appropriate baseline model in order to put the model’s performance in context. We consider two such models: a random guessing model and a mode guessing model. Both types of models can be derived from the proportions of observations from each class in the training data.

In the random guessing model, we make predictions by randomly sampling from a multinomial distribution with class probabilities determined from those proportions. Note that this model is “naive” in the sense that it ignores the input features entirely. If a predictive model fails to outperform this baseline, then one can conclude that either the features contain no useful information about the variable that is being predicted, or the model is misspecified and is not appropriate for the dataset.

In the mode guessing model, we simply predict the most common class from the training data. That is analogous to the heuristic that a human might use in the absence of outside information. In the context of pain, when asked to estimate pain without additional data, a clinician might predict that each patient is experiencing the level of pain that is typical among similar patients or that the pain has not changed since the last time the pain was reported. A predictive model that fails to outperform such a model would have limited usefulness in a clinical setting.

Since 10-fold cross-validation involves random assignment of patients to training and testing folds, the predictions of both of these naive models can be viewed as random variables. One can use bootstrapping to simulate this assignment and prediction process in order to estimate the probability distributions for those predictions as well as any relevant performance metrics (such as those discussed in Section 4.3). Given these distributions and the performance of a trained model, it is straightforward to estimate the probability that the naive model outperforms the trained model according to a given metric. These probabilities are analogous to the so-called *p*-values used in hypothesis testing. In other words, we can use these *p*-values to test the null hypothesis that a naive model performs as well as or better than the trained model. The *p*-values reported in Section 2 were computed using this approach. According to convention, *p*-values less than *α* = 0.05 are deemed statistically significant at the 0.05 level, and constitute evidence that the null hypothesis can be rejected in favor of the alternative. In this context, *p* < 0.05 constitutes statistically significant evidence that the trained model outperforms the naive model.

#### 4.2.4 Alternative models

One limitation of the classification models presented here is that the pain scores are binned into unordered classes. As far as the classifier is concerned, pain scores of 6 and 7 are equally distinct as pain scores of 4 and 7 since both pairs correspond to the same pair of classes: medium and high. Similarly, there is no requirement that medium and high pain classes should be more similar than low and high classes in the model. The models are free to discover this ordinality from the data, but the ordinality is not imposed a priori.

One way to take the ordinality of pain into account is to interpret this as a regression problem rather than a classification problem. One could attempt to predict the pain, the deviation from the median pain, or the change in pain directly instead of assigning those continuous quantities to discrete classes. Standard methods such as linear regression or linear Gaussian state space models (the continuous analog of the HMMs discussed above) could be used to uncover the dependencies between the physiological measures and these numerical pain values. Unfortunately, these methods either operate under the assumption that these dependencies are linear or they require assumptions about the form of any nonlinearities. In preliminary testing, we found that these linear models underperformed their discrete counterparts and therefore we do not discuss them here.

### 4.3 Evaluation metrics

In Section 4.2, we presented a variety of classification models for the evolution of pain. Given a sequence of emissions, these models can be used to produce probabilistic predictions for the corresponding pain categories. We now discuss various metrics used to compare the performance of these models.

The most natural tool for describing the performance of a classifier is the *confusion matrix*. Given an *N*-class classification problem, a confusion matrix is an *N* × *N* matrix with entry in row *i* and column *j* indicating the number of examples in the test set for which the true class was *i* and the predicted class was *j*. Unfortunately, there is no straightforward way to compare confusion matrices since they are multi-dimensional. Researchers often attempt to condense that information into a single metric allowing for direct comparisons. For example, given a confusion matrix *C*, the diagonal entries correspond to correctly classified observations, and one can therefore compute the accuracy by computing the proportion of correct predictions out of the total number of predictions.

Unfortunately, accuracy is not necessarily a good measure of performance. In classification problems with imbalanced class-sizes, naive models like the mode-guessing model can produce a high degree of accuracy despite the fact that their predictions are useless. This is analogous to a weatherman who never forecasts rain: although the forecast may be correct 90% of the time, it does not provide any useful information.

Alternatives to accuracy include the *precision* and *recall* which measure the number of true positives divided by the total number positive predictions and the number of true positives divided by the number of positive examples respectively. Precision is therefore the more useful measure when one is primarily concerned with avoiding false positives and recall is the more useful measure when one is primarily concerned with avoiding false negatives. Both metrics are bounded between zero and one with zero corresponding to a classifier that is always wrong and one corresponding to a perfect classifier. For *N*-class classification problems, one can compute each metric *N* times, once for each “positive” class and then average the results to obtain a single number. When the classes are weighted equally during the averaging, the metrics are referred to as the *macro* averaged precision and recall. When the classes are weighted in proportion to the number of observations in each class, the metrics are referred to as the *weighted* average precision and recall. Both averaged metrics are difficult to interpret without knowing the sizes of the classes.

The *F*_1_ *score* is the harmonic mean of the precision and recall and is a way to combine both quantities into a single metric. Like precision and recall, it is possible to compute macro average and weighted average *F*_1_ scores by averaging over the number of classes. Unfortunately, this can lead to counterintuitive results. For example, it is possible to have a macro averaged *F*_1_ score that is lower than both the precision and recall. Consider a three class problem with 10 examples from each class and consider a classifier with predictions that are biased toward class 2 due to an overabundance of examples from class 2 in the training data. Suppose that this classifier produces the confusion matrix shown in Table 7. The single class precisions are given by 1/1, 1/1, and 10/28 and the single class recall values are given by 1/10, 1/10, and 10/10 for classes 0, 1, and 2 respectively. This means the *F*_1_ scores are given by 2/11, 2/11, and 10/19. These results can be averaged across classes (in this case macro and weighted averaging are equivalent) yielding averaged scores of 11/14 ≈ 0.786, 2/5 = 0.4 and 62/209 ≈ 0.297 for precision, recall and *F*_1_ score respectively. In other words, *F*_1_ score is lower than both the precision and the recall despite the fact that it is the harmonic mean of those quantities when applied to each class. On the other hand, if we take the harmonic mean of the averaged precision and recall, we obtain 0.530 which is in between precision and recall.

**Table 7:** Confusion matrix for a hypothetical classifier. In this table, rows indicate the true class and columns indicate the predicted class. The entries in the table describe the number of observations of each type. When computing the *F*_1_ score for this classifier, one finds that, counterintuitively, the averaged result is smaller than both the precision and the recall.

	0	1	2
0	1	0	9
1	0	1	9
2	0	0	10

Because average *F*_1_ scores are popular multi-class classification metrics, we include the macro-averaged *F*_1_ score in Tables 2–4 for the sake of completeness. However, due to these counter-intuitive properties, we do not consider these metrics to be particularly informative in the context of pain estimation.

Up to this point, all of the metrics we have discussed are based on the confusion matrix. In other words, the metric is computed from the predictions rather than the class probabilities. These predictions are obtained by selecting the class with the highest posterior probability. Unfortunately, in problems with imbalanced classes that are not well-separated, the priors (related to class frequencies of the classes) can dominate the likelihoods so that the posterior probability is always (or almost always) largest for the most common class and the classifier will never (or rarely) predict the minority classes. This does not necessarily mean that the posterior probabilities are uninformative. For example, suppose a patient usually has high pain with probability 0.8 and low pain with probability 0.1. If at a particular point in time, the posterior probabilities for high and low pain change to 0.55 and 0.3 respectively, then that is a sign that the patient’s condition may have improved and that the model is detecting evidence for that change. Metrics that are based on the confusion matrix cannot detect these small changes since the “High” pain class has the highest posterior probability in both cases.

The volume under the receiver operating characteristic surface (VUS) and its binary analog, the area under the receiver operating characteristic curve (AUC), use the posterior probabilities directly instead of the confusion matrix. For example, in a binary classifier one can use a threshold on the posterior probability for the positive class to determine whether to label the example as positive or negative. When this threshold is close to zero, the true positive rate (TPR) will be high, but the false positive rate (FPR) will also be high. One can reduce the false positive rate by increasing the threshold, but this may decrease the true positive rate as well. As the threshold varies, the TPR and FPR trace out a parametric curve (FPR,TPR) = (0,0) to (FPR,TPR) = (1,1). A useful classifier will possess a particular threshold with a low FPR and a high TPR. The area under this curve therefore provides a useful metric for measuring classifier performance.

The VUS is a generalization of the AUC to multi-class problems [15]. Direct computation of the VUS for an *n*-class classification problem involves integrating over a surface in *n*(*n* − 1) dimensions. This can be computationally intensive, so VUS is usually approximated using certain heuristics. The approach we use is motivated by reference [16], where the authors provide a concise interpretation of the VUS. They demonstrate that the VUS is equal to the accuracy obtained in a forced-choice experiment where the classifier is presented with examples from each class and asked to assign labels to each. This procedure can be implemented as follows:

1. *Initialize two counters. Total = 0 and TotalCorrect = 0*.
2. *Select one example from each class from the testing set*.
3. *Using the posterior class probabilities, attempt to assign labels to each of these examples*. Note that this essentially a matching problem since we know that there must be an example from each class. We used decision rule III from [15] for the matching procedure.
4. *If all of the labels are correct, then increment TotalCorrect and Total. Otherwise, increment Total only*.
5. *Repeat steps 2 through 4 until all combinations of examples from each class have been exhausted*.
6. *The accuracy in this task, and therefore the VUS score, of this classifier is given by TotalCorrect/Total*.

Note that a trivial classifier that guesses randomly will have an accuracy of 1/*n*! where *n* is the number of classes. This means that, in binary classification problems, nontrivial models should exceed a baseline VUS of 0.5, and in three-class classification problems such as those discussed here, the VUS score should exceed 0.167.

The number of combinations of examples from the classes grows quickly with the size of the dataset and the number of classes making this procedure impractical for even moderately sized datasets. Fortunately, one can obtain a satisfactory approximation by using random samples from each class rather than deterministically exploring every combination of examples from each class. We found 1000 samples to be sufficient for obtaining satisfactory estimates for the VUS.

Out of all the metrics presented here, we believe that the VUS is the most meaningful for the pain estimation problem due to its robustness to class imbalance and because of its ability to detect whether the posterior probabilities contain useful information for distinguishing between classes. According to this metric, both HMMs and GNB classifiers provide a significant improvment over null models despite the fact that the trivial mode classifier has higher accuracy.

## Supporting information

S1 Fig Normalized features and median referenced pain scores. These scatterplots depict the correspondence between the normalized physiological features and relative pain scores for all patients. Red curves display a linear fit. Individually, the correlations between these features and the (median-referenced) pain scores are weak. The correlations are 0.039, 0.022, 0.094, -0.031, 0.037, 0.028 respectively for Resp, Temp, Pulse SpO2, Systol and Diastol. However, the evidence that the slope differs from zero is weak and is significant at the *p* = 0.05 level for only Resp and Pulse. However, the performance of the classification models suggests that when all six features are used in tandem, a clearer signal can be extracted.
(EPS)

S2 Fig Sample time series. These plots display the temporal evolution of the pain scores and vitals for two representative patients. The absence of simple trends in the pain scores suggests that a probabilistic temporal model such as a (hidden) Markov model is more appropriate for describing the dynamics than a deterministic model.
(EPS)

S1 Table Confusion matrices for the HMM model. Rows correspond to the true class and columns correspond to the predicted class. The values represent the number of observations with the corresponding true and predicted classes. The confusion matrices are aggregated over the ten testing folds. The accuracy is high for the largest class in each classification problem, but lower for minority classes. The success of the models according to alternative metrics like VUS suggests that the posterior probabilities are more informative than the predictions themselves.
(EPS)

S2 Table Confusion matrices for the GNB model. Rows correspond to the true class and columns correspond to the predicted class. The values represent the number of observations with the corresponding true and predicted classes. The confusion matrices are aggregated over the ten testing folds. The accuracy is high for the largest class in each classification problem, but lower for minority classes.
(EPS)

S3 Table Transition matrices for HMM models. Rows correspond to the source class and columns correspond to the target class. Values indicate the probability of transitioning between source and target classes at each time step (2 hour window). These values were obtained by averaging over 10 models resulting from the 10 folds used in cross-validation. The transition matrices reveal plausible trends in the learned dynamics. Both the direct and median-referenced models show evidence of auto-correlation, i.e., they indicate that the next state is correlated with the current state. The difference model shows regression to the mean, i.e., it indicates that pain increases are more likely to be followed by decreases and vice versa.
(EPS)

S4 Table Emission parameters for HMM models. Rows indicate the pain class and columns indicate the emission type. The values displayed include the mean ± the standard deviation. The emission parameter tables show that the variance between classes is much smaller than the variance within classes. This means that, on its own, each emission variable would be insufficient for classifying the pain. However, when used in tandem, these physiological measures can provide a meaningful signal as evidenced by the statistically significant improvement in VUS. For most of the physiological measures, there is no clear trend in the means across classes. This can be attributed to the confounding variable of medication. In a controlled setting, as pain goes up, one would expect the physiological measures (with the exception of SpO2) to increase as well. However, the patients studied here are more likely to receive pain medication when the pain is high. These medications tend to have the opposite effect, causing the physiological measures to decrease. Because of these opposing effects, it is unclear whether one should expect high pain to be associated with higher physiological measures or lower.
(EPS)

S5 Table Emission parameters for GNB models. Rows indicate the pain class and columns indicate the emission type. The values displayed include the mean ± the standard deviation.
(EPS)
